# Making a dead woman pregnant? A critique of the thought experiment of Anna Smajdor

**DOI:** 10.1007/s11017-023-09642-2

**Published:** 2023-08-22

**Authors:** Erwin J.O. Kompanje, Jelle L. Epker

**Affiliations:** Department of Intensive Care, Erasmus MC University Medical Center, Rotterdam, the Netherlands

**Keywords:** Brain death, Whole body gestational donation, IVF, Surrogacy

## Abstract

In a thought-provoking article – or how she herself named it, ‘a thought experiment’ – the philosopher-medical ethicist Anna Smajdor analyzed in this journal the idea of whole-body gestational donation (WBGD) in brain-dead female patients, as an alternative means of gestation for prospective women who cannot or prefer not to become pregnant themselves. We have serious legal, economical, medical and ethical concerns about this proposal. First, consent for eight months of ICU treatment can never be assumed to be derived from consent for post-mortem organ donation; these two are of an incomparable and entirely different medical and ethical order. Moreover, the brain-dead woman is very likely to be medically unfit for high-tech surrogacy and the brain-dead state poses a high risk for deficient embryo/fetal development. Second, from a scarcity perspective, occupying an ICU bed for eight months appears to be unjust. The costs for eight months of ICU treatment are far too high compared to the costs of surrogacy for a living, selected, and healthy woman. Neither insurance companies nor prospective parents will want to pay these exceptionally high costs for a dead woman if a living surrogate mother can be hired for a considerably lower amount. Third, there is an increased risk for harm of the child to be in WBGD. And finally, WBGD risks violating the brain-dead woman’s dignity and harming the interests of her loved ones. In short, there is simply no need for brain-dead women as surrogates.

*‘So, act that you treat humanity, whether in your own person or in the person of any other, always at the same time as an end, never merely as a means’*.

Immanuel Kant, *Kritik der reinen Vernuft*, 1785; p. 429.

## Introduction

In a thought-provoking article – or how she herself named it, ‘a thought experiment’ – the philosopher-medical ethicist Anna Smajdor analyzed the idea of whole-body gestational donation (WBGD) in brain-dead female patients in the intensive care unit (ICU) as an alternative means of gestation for prospective women who cannot or prefer not to become pregnant themselves [1]. Normally, a surrogate mother is a physically and mentally healthy pre-menopausal woman who intentionally becomes pregnant on behalf of the intended parents. After birth, the surrogate mother gives up the child to these parents. This is different from Smajdor’s experiment which considers the use of mechanically ventilated bodies of brain-dead women rather than the bodies of physically and psychologically healthy women. The body of the brain-dead woman could, according to Smajdor, serve as a surrogate organism with intact bodily homeostasis which could gestate an embryo for up to nine months. Smajdor followed the article by Rosalie Ber who suggested the same practice for female patients in Persistent Vegetative State (PVS) in 2000 [2]. We have, however, serious medical, economic, and ethical concerns about this proposal.

## Scarcity

‘Desperate times call for desperate measures’ is a phrase which most probably originates from Hippocrates and was later used in 1500 by Desiderius Erasmus with the phrase *‘Malo nodo, malus quaerendus cuneus*’ (translation: A harsh knot is not to be attempted to be cut with a fine tool: it can only be overcome by the application of a strong wedge) [3]. This is what Anna Smajdor must have thought, we suppose, when she proposed to use brain-dead women as surrogate mothers. Indeed, scarcity sometimes justifies desperate measures. But is there such a scarcity on surrogates that such an extreme measure is reasonable?

Traditional surrogacy may be altruistic (not-for-profit) or the gestational carrier may be paid (commercial or compensated surrogacy). The number of parents having a baby using a surrogate mother in England and Wales rose from 117 in 2011 to 413 in 2020. There is, at this moment – in countries in which surrogacy is allowed – no scarcity of potential surrogate mothers. Natalie Gamble, director of *Brilliant Beginnings* – a non-profit organization which matches surrogates and intended parents – said in September 2021 to the BBC: ‘We get around 100 enquiries a month from women who want to be surrogates and that’s risen considerably in the past few years’ [4]. There was a fourfold increase in the number of surrogacies in the USA between 1999 and 2013 and it is still on the rise. Between 2006 and 2010, there was a 1.000% increase in the market of international surrogacy [5]. There are reports of international surrogacy agencies claiming a growth of 6.000% in 12 years [5]. National legislation concerning surrogacy varies substantially, leaving most countries unregulated. Surrogacy in Europe is allowed – or at least not banned – in Albania, Armenia, Belarus, Belgium, Cyprus, Czech Republic, Georgia, Greece, Ireland, Macedonia, Portugal, Romania, Russia, the Netherlands, United Kingdom and Ukraine [5]. Although there was a 60% drop in potential surrogacies due to the COVID-19 pandemic, there is still not a shortage such that desperate measures – like using brain-dead patients in the ICU as gestational surrogates – should be considered. The Asia-Pacific region has presented lucrative opportunities for key players operating in the surrogacy market. For example, surrogacy in India is cheaper compared to more developed countries, thereby contributing to market growth of surrogacy there and an overall decline in costs [6]. However, as surrogacy is now however banned in India, Georgia is emerging as an alternative cheap destination [7].

Taking this into consideration, we are by no means convinced that there is a great need to look for sources outside of traditional surrogacies at this moment. The number of potential surrogacies is sufficient to meet the growing demand. Thus, the current time is not desperate with regard to surrogacy that would justify in any way Smajdor’s proposed measure.

## High-tech surrogacy

Two forms of surrogacy can be distinguished from each other: traditional (low-tech) surrogacy and gestational (high-tech) surrogacy. In traditional surrogacy, a pregnancy is achieved in the surrogate mother through artificial insemination. The surrogate mother is the genetic mother and the intended father is the genetic father of the embryo. In gestational surrogacy, the eggs of the intended mother are fertilized in the laboratory with the sperm of the intended father. One embryo is then placed in the uterus of the surrogate mother via in vitro fertilization (IVF). After birth, the child is transferred to the intended parents. In the Netherlands, only one University Hospital facilitates gestational surrogacy. The surrogate mother must meet several strict conditions, such as: the surrogate mother must have been pregnant before without any complications; the surrogate mother must be both physically and mentally healthy; there must be no indication of an increased risk of complications during pregnancy; and the surrogate mother must be between 25 and 43 years old at the time of registration.

According to this model, the gestational surrogates are, with good reason, selected very strictly because a high-tech regulated pregnancy has – unsurprisingly – a very high failure rate, even if all criteria are met. Thus, we hypothesize that a brain-dead woman in an ICU – being kept for many months on a ventilator – can *never* meet these strict selection criteria for several reasons, as we discuss below.

## The brain-dead patients

Brain death has become a rare outcome within intensive care medicine. Among 4248 patients who died between 1999 and 2000 in European ICUs, 330 (7.8%, regional differences between 3.2% in Northern Europe and 12.4% in Southern Europe) deaths were diagnosed as brain dead [8]. In the United States, brain death accounts for 2% of all hospital deaths and primarily consists of male patients with a mean age of 47.8 years [9]. The causes of brain death are limited: traumatic brain injury (TBI), subarachnoid hemorrhage (SAH), cerebral hemorrhage (ICH), and circulatory arrest accounting for more than 80% of the cases. An analysis of 71 published series of brain-dead patients (n = 6317) showed that a (aneurysmal) SAH, TBI and ICH preceded brain death in 83% of cases [8]. In many countries, aneurysmal SAH is the leading cause of brain death in the ICU [8]. There is an overrepresentation in male patients with TBI and an overrepresentation in female patients with SAH. Most of the women suffering from a SAH are postmenopausal. Among 145 patients admitted with a SAH who died on the ICU, the median age was 69 years (range 40–86 years); 55% of which suffered from drug treated hypertension preceding the SAH [8]. However, a SAH is a rare form of stroke, accounting for approximately 1–7% of all strokes [8]. SAH have two common risks factors: cigarette smoking and hypertension. In several population-based cohort studies, 70 to 75% of patients with SAH have a history of smoking, and 50–60% are current smokers. Hypertension is the most common comorbidity in patients admitted with a SAH. The pharmacologic treatment of hypertension reduces the risk of stroke, including SAH and ICH, which has been confirmed in many trials [10].

Now, when considering the criteria for a woman to qualify as a gestational surrogate, almost all brain-dead women will *not* qualify. Most of them will be too old, if not already menopausal. Moreover, they are most likely not physically healthy (e.g., high percentage of arterial hypertension, long history of smoking, etc.). These comorbidities are – generally – not strictly a contraindication for organ donation, but would certainly be a contraindication for a healthy pregnancy, let alone high-tech surrogacy. A brain-dead surrogacy – or WBGD – in this sense, carries an undesirable high-risk of failure in the surrogacy.

Is it realistic to say that a brain-dead patient can be treated in an ICU in optimal physical condition for about eight months? This assessment is conditional before the precious embryo can be placed in the womb of the brain-dead woman. Although many brain-dead patients are hemodynamically unstable and will eventually face irreversible circulatory arrest (despite intensive care support), some are remarkably stable after the brain death determination. The assumption that all brain-dead patients only remain stable for a few hours can be falsified. As early as 1998, Alan Shewmon published a meta-analysis on cases of ‘chronic brain death’ [11]. In 2006, Susan Repertinger et al. [12] published a report on the brain autopsy of a boy, “T.K.”, who was determined brain dead at an age of 4 1/2 years and who survived on life support for an additional two decades. At autopsy, his brain was found to be completely destructed consisting of a calcified shell containing grumous material without any recognizable anatomical brain structures. No posterior fossa structures (e.g., brain stem, cerebellum, circle of Willis, or cranial nerves) were detectable. Whole brain death was complete and pertinent. The elapsed time gave rise to complete calcification and afunctional destruction of his entire cranial contents. The illustrations accompanying the article clearly show this.

It is conceivable, then, that *some* pregnant brain-dead women could be kept stable in intensive care for weeks or months to allow the fetus to grow in the womb, despite the fact that the woman’s entire brain has been destroyed. However, we think that prolonging the state of brain death for a very long time is never in the best interest of the patient. In the case of the 4 1/2-year-old boy, his parents – as his surrogate decision makers – had decided to renew for the duration of twenty years in a specialized care facility. This type of consent cannot be assumed in the case of adult patients. This is the same for WBGD; without explicit written prior consent of the patient (or consent of the relatives of the patient), it is simply not legal. Smajdor states that it does not matter whether mechanical ventilation and intensive care treatment are extended for hours, days, or months because the goal of organ donation or WBGD is helping others, and this end justifies the means. However, this is only partially true. The period of ventilation in brain-dead patients for the means of organ donation is rarely more than one or two days. After consent is obtained, the brain-dead patient is stabilized in the ICU and then organ matching is performed; afterward, the donation process is started as soon as possible. So, firstly, it is problematic to ignore the medically and ethically disproportionate ventilatory and hemodynamic treatments in a dead patient. Secondly, because a longer stay in the ICU increases the risk of additional complications (like decubitus ulcers and ventilator or IV-line associated infections), the quality of the organs intended to be donated is jeopardized. Moreover, while organs are scarce and simply unable to be retrieved in any other way, surrogate pregnancy is not scare and available in other ways as explained above. Thus, to say that keeping a brain-dead woman in the ICU on a ventilator for 2 days for an organ donation procedure has the same impact for the patient, the society, and the family as compared with keeping her body alive for nine months for WBGD is a statement that falls short completely. Other practical, economic, and ethical considerations, like costs, insurance, patient dignity, justice, scarcity, prenatal physical stress, compassion, and explicit consent, makes this comparison even more convincingly incorrect. We now turn to examining these considerations.

## Costs and insurance

In Anna Smadjor’s thought experiment, a brain-dead woman is in the ICU and will have to stay there on life support and mechanical ventilation for about eight to nine months after the embryo is placed in the womb. It will first have to be determined whether the embryo transfer is successful; after all, in normal and optimal situations for *standard* in vitro implantations, many attempts fail. The attempt to transfer an embryo to the uterus of a brain-dead woman in an ICU is far from optimal and far from normal. How many attempts of inserting an embryo into the uterus of the brain-dead women are acceptable in this situation?

In Belgium and the Netherlands, the basic cost for a one day stay in the ICU is approximately between € 1545 and € 3221 (median €2160) and the nursing cost between €496 and €1229 (median €789) making daily costs approximately €3000 [13]. In the United States daily ICU-costs were greatest on the first day ($7728 - $8509), decreased on day 2 ($3872 - $4223) and became stable from day 3 forward ($3436 - $3550) [14]. In Australia the mean cost per patient bed-day was $4375 [15]. With complications, the costs will be higher. On average, an ICU patient stays 7 days in the ICU. So, a general ICU admission costs approximately $15.120 and $30.625. Accordingly, an eight month stay in the ICU for WBGD would cost approximately €480.000 (Europe), $795.000 (USA) and $980.000 (Australia). To think about it differently, for the price of one WBGD admission, another 33 patients could be treated in the ICU.

The costs for a healthy and living gestational surrogacy are approximately €25,000 - €30,000. The costs for IVF are approximately €5000 - €6000. In the USA the costs will be between $30.000 and $40.000 [16]. In the Netherlands, after legal securities have been arranged, high-tech surrogacy is expected to be reimbursed through basic insurance from 2024 onwards. By then, health insurers will have to adjust their policy conditions accordingly. A brain-dead woman, however, is legally dead; after which her personal rights and obligations will cease. Her own health insurance will therefore stop and will assuredly not reimburse the costs for eight months of ICU treatment. Moreover, the intended parents will probably not be willing to pay this large amount of money either, because surrogacy with a healthy, selected, and living surrogacy is much cheaper and involves much less risk. To put it another way, twenty well selected healthy surrogacies cost the same amount of money as one brain-dead surrogacy. Therefore, deciding which surrogacy (dead or alive) to choose will not be very difficult for anyone.

## Consent

Both in PVS and brain death, explicit prior written consent for WGBD should be required. Both Ber and Smadjor state that prior written consent in cases of PVS will never actually be available given the fact that no one will think about this scenario in advance, and the same is true for brain-dead patients. However, Smadjor does see these possibilities for brain-dead patients, because there is already a registration system for post-mortem organ donation. Individuals could give explicit consent for WGBD in advance through the same registration system, or consent could be presumed if no objection is registered for postmortem use of the body. She concluded that if current consent protocols are acceptable for organ donation based on a minimal information perspective, they should also be acceptable for WGBD, perhaps with some additional informational campaigns.

Although there are registration systems in different countries that theoretically could serve as a source of this type of consent, one must not forget what these registration systems represent: namely, organ- and tissue donation registries, no less and no more. They simply do not include, in any way, something like the whole-body donation that would be necessary for effectuation of WGBD.

Suggesting that WBGD could be organized by introduction of a “simple” opt-out within the current systems of organ donation registries is not congruent with the real world; its introduction would violate virtually all rules of ethical communication and is therefore wholly unacceptable. If all relevant women (18–45 years old, proven fertile, and in good health) in a country are to be reached with a “sign out” campaign, it will definitely not be a simple campaign that only addresses “some additional information”, and it will definitely not be cheap. Moreover, recent history (e.g., the Jahi and Archie cases) has shown that brain death can be extremely sensitive. Thus, any change in a registration system like this, even if it looks only minor in the eye of the beholder, can cause a major stir in the general public, compromising public trust and thereby lowering registration rates for organ donation. It is worth asking – when all these hurdles have been taken, who exactly are they for?

## Justice and scarcity of ICU beds

The COVID-19 pandemic demonstrated evidence of the limited capacity of health systems to respond to an increased demand. The demand on (staffed) ICU beds has already exceeded capacity for many years in many countries but became even more visible during the COVID-19 pandemic. In response, triage protocols have been made in the face of excessive demand on ICU capacity. The question whether someone should be ventilated on an ICU should be restricted to the individual patient, but the protocols address the question of who should be ventilated assuming a choice between two patients [17]. Intensive care can be defined as a service for patients with potentially recoverable conditions who can benefit from more detailed observation and invasive treatment than can safely be provided in general wards or high dependency areas. Thus, patients who are too well to benefit or those with no hope of recovering to an acceptable quality of life should not be admitted [18]. For patients without the possibility of an acceptable recovery, the treatment will have to be terminated. If the patient dies, the treatment ends.

The exception is the brain-dead patient who, after the declaration of death, is ventilated and treated for several hours, and only with consent, until the organs are removed for transplantation. Related is the extremely rare case of a pregnant brain-dead woman, when in exceptional situations it can be decided to continue mechanical ventilation and treatment for several weeks or months in order to allow the fetus to grow until the age of viability. Accordingly, to attempt to treat a brain-dead woman for more than eight months and induce the pregnancy in the ICU is an inappropriate use of already scarce ICU beds and staff. Therefore, WBGD is very unjust towards critically ill patients who would possibly benefit from admission to an ICU but for whom no bed is available. Furthermore, as mentioned above, who will bear the inappropriate high costs of treating and ventilating a deceased person for such a long period?

## Prenatal maternal stress

Intrauterine exposure to medicinal and social drugs, along with mental stress of the pregnant woman may cause structural and/or functional developmental deficits that often result in life-long physical and mental handicaps. Potentially harmful drug-drug interactions are described in maternal intensive care [19] as well as physiologic and pharmacokinetic changes in pregnancy [20]. This is also true for nutritional deficiency and shortage [21, 22]. Many patients in the ICU have lower plasma levels of essential vitamins and micronutrients. Even progressive enteral tube feeding – containing vitamins and trace elements – does not normalize plasma levels in the first few weeks in the ICU [23]. Because the embryo/fetus in a brain-dead woman will be exposed to the many negative effects of the ICU stay (administration of pharmaceuticals, possible deficiency in hormones, lack of adequate vitamins and micronutrients, the treatment of infections, pulmonary care for a mechanically ventilated patient, abnormal noise pollution, etc.) without normal interaction with the mother for eight months, the developing embryo/fetus in the womb of the brain-dead woman will have a high risk of developmental deficits. Why should a couple who have chosen or been advised to a high-tech surrogacy take such a high risk and at such high costs? Why should they take a risk that they would not have when choosing a healthy and strictly selected living woman as surrogate? We cannot think of any reason. In a risk-benefit assessment for the fetus, WGBD as proposed by Anna Smajdor will fail as an acceptable benefit.

## The (ab)use of a dead woman

Anna Smajdor mentions, without reference to the source, that ‘WBGD involves treating the patient’s dead body as a means to an end, rather than an end in itself’ [1]. The source of this statement is the Categorial imperative by the German philosopher Immanuel Kant (1724–1804). This is the most important philosophical concept concerning humanity in his deontological moral philosophy. According to Kant, people are ‘ends in themselves’ and have intrinsic value, not mere instrumental value. Treating patients solely as a means (e.g., treating patients solely as an instrument to obtain something for oneself, such as organs for transplantation or a living womb to gesture a fetus), fails Kant’s test [24]. One could value a brain-dead patient as an ‘organism’ and a ‘non-person’ as Lazaridis has suggested, escaping the rules of the Categorical Imperative as there is no ‘rational agent’ to respect [25]. Can physicians, nurses, and relatives of a brain-dead woman view her as a non-person, an organism, a ventilated cadaver for eight months long? It will be very difficult, if not impossible, to see a pregnant brain-dead woman as such. As the Categorical Imperative is categorical and thus applies to everyone without exception, it is not applicable to a non-person or cadaver but is applicable to a person. Does one see the brain-dead woman – when a fetus grows inside her – as a living person or as a deceased person? Seeing her as a corpse in case of organ donation and as a living person in case of WGBD creates a strange legal paradox that will feed opponents of “brain death as equal to death” and thereby unintentionally but still potentially severely harm “the brain-dead as dead” medical consensus for organ donation purposes in the public opinion.

## Dignity and compassion

Human death is determined based on cardiopulmonary criteria (the heart no longer circulates blood and there is no longer oxygen-carbon dioxide exchange through the lungs) or on the basis of neurological criteria (the functions of the brain that are essential for life are irreversibly lost). The latter is considered as “death” in almost all countries of the world and is supported by laws and protocols. Of course, some will not see “the brain-dead” as “dead”, making brain-death controversial, but there is general consensus within the medical community. However, because there are still signs of “biological life” (only there because of a medical intervention with the ventilator), it is difficult for some to consider the ventilated body of a brain-dead patient as “deceased”. Compared to a deceased person who died after a cardiac arrest, the brain-dead patient has many “signs of life”. That complicates the handling of a brain-dead patient as a dead body. The fact is that patients who have been declared “brain-dead” on the basis of the established criteria have never shown any full recovery of consciousness. Refer to the case described by Susan Repertinger et al. [12]; so called “awakened” brain-dead patients never have met the very strict criteria in most European countries when they were declared brain-dead and have therefore never been brain-dead at all. In the context of organ donation, brain death is widely accepted in the world. A brain-dead patient resembles in many ways a deeply comatose patient. One handles a deeply comatose patient with respect and dignity. The moral status of a patient or a dead human body is, however, different. We assume that human value and dignity comes to an end with death. But we also have strong intuitions about how to handle the dead with respect. For the relatives, the existence of their loved one does not end with death. They still treat the bodies of their beloved deceased with the same respect and dignity as when they were alive. Because they are dead, however, there is the risk that others can treat them without dignity. They are therefore vulnerable to dignity violations.

Dying with dignity is highly valued. Everyone has a right to die with dignity, comfortably, without pain and distress. In palliative care for the terminally ill, this is pursued professionally. In some countries, a dignified death can be achieved by the deliberate termination of life as requested by the patient. “Letting someone die in peace” is an internationally appreciated concept. In 1995, the German anatomist Gunther von Hagens premiered Body Worlds, gathering and exhibiting plasticated human bodies in lifelike poses. A medical ethicist, Carol Taylor, argued: ‘My major objection stems from the belief that there is an innate dignity to humans that extend to our bodies…They were denied a proper burial and did not give consent to be on public display’ [26].

We think that the same value of dignity should be given to brain-dead women. Give the brain-dead woman’s loved-ones space to begin their mourning over the loss. Cease the means that obscure the real death. Let them tell all who knew and loved the woman that she is dead and that they can pay their respects at the funeral. Let them see the corpse to convince them of the death of the woman. Do not let them tell their family and friends that their loved one is dead but that the funeral will not take place for another year. That is far from death with dignity.

## Conclusion

Anna Smajdor named her article a ‘thought experiment’ and we would best like to leave it that way, or maybe call it an *idée fixe* since her proposal faces, as we have argued, significant ethical, legal, and economic obstacles. Ultimately, there is no need for brain-dead women as surrogates. Consent for eight months of ICU treatment can never be assumed to be derived from consent for post-mortem organ donation; the two are of an incomparable and entirely different order. The brain-dead woman will generally be medically unfit for high-tech surrogacy and WBGD poses a high risk for deficient embryo/fetal development. From a scarcity perspective, occupying an ICU bed for eight months is unjust. The costs for eight months of ICU treatment are far too high compared to the costs of surrogacy with a selected and healthy woman. Insurance companies will not want to pay these exceptionally high costs for a dead woman, and the prospective parents will not want to pay either if they a living surrogate mother can be hired for a considerably lower amount. Even if payment is settled in one way or the other, the amount of money spent is so disproportionately high that spending it is unethical compared to the costs of treatment for a normal ICU patient. Finally, there is the risk of violating the brain-dead woman’s dignity and harming the interests of her loved ones. Treat a person, even if she is dead, as an end in herself, not as a means to an end outside her.
